# Well-Differentiated Intraosseous Osteosarcoma in the Sacrum: A Case Report

**DOI:** 10.5812/iranjradiol.13172

**Published:** 2013-08-30

**Authors:** Wang Liuhong, Zhang Minming

**Affiliations:** 1 Department of Radiology, the Second Affiliated Hospital, Zhejiang University, School of Medicine, Hangzhou, China

**Keywords:** Sacrum, Osteosarcoma, Sarcoma, Alveolar Soft Part

## Abstract

Hereby we report a case of well-differentiated intraosseous osteosarcoma in the sacrum. A 32-year-old woman was admitted to our hospital with a low echo-level mass in the pelvis searched by ultrasound in a routine physical examination. Radiographically, the mass was misdiagnosed as a benign bony tumor originating from the sacrum. The tumor was completely resected and pathological diagnosis was intraosseous well-differentiated osteosarcoma. Twelve months after operation, the patient was well and there was no evidence of recurrence and distal metastasis. This is a peculiar case of well-differentiated osteosarcoma involving an unusual site of the sacrum. The radiographic appearance and the differential diagnosis are discussed. We consider that dense trabeculated-like bone within an intraosseous solid mass might be suggestive of a well-differentiated osteosarcoma that was valuable in guiding the treatment and prediction of its prognosis. Well-differentiated osteosarcoma, although malignant, may be mistaken for a benign condition. Local excision has almost always been associated with recurrence. For this case, the patient had a wide excision and had no recurrence and metastasis. Therefore, it is very important to identify the radiological features and to distinguish this tumor from benign lesions and high-grade osteosarcomas before operation.

## 1. Introduction

Well differentiated osteosarcoma of the bone is rare, accounting for approximately 1.2% of all osteosarcomas (1) and was first described by Unni et al. (2). This tumor, although malignant, is indolent and has a relatively good prognosis. However, it is commonly misdiagnosed, both pathologically and radiologically as a benign lesion. The distal femur is the most frequent involvement site (3). However, low-grade osteosarcoma of the sacrum has not previously been reported. In this report, we have reported a well-differentiated osteosarcoma involving the sacrum of a 32-year-old woman. The lesion was located in number 3-5 sacrum segments (S3-S5), and showed an expansile mass expanding anterior into the pelvis. We report this unusual location and the radiological and histological findings of the well-differentiated intraosseous osteosarcoma.

## 2. Case Presentation

**Figure 1.**
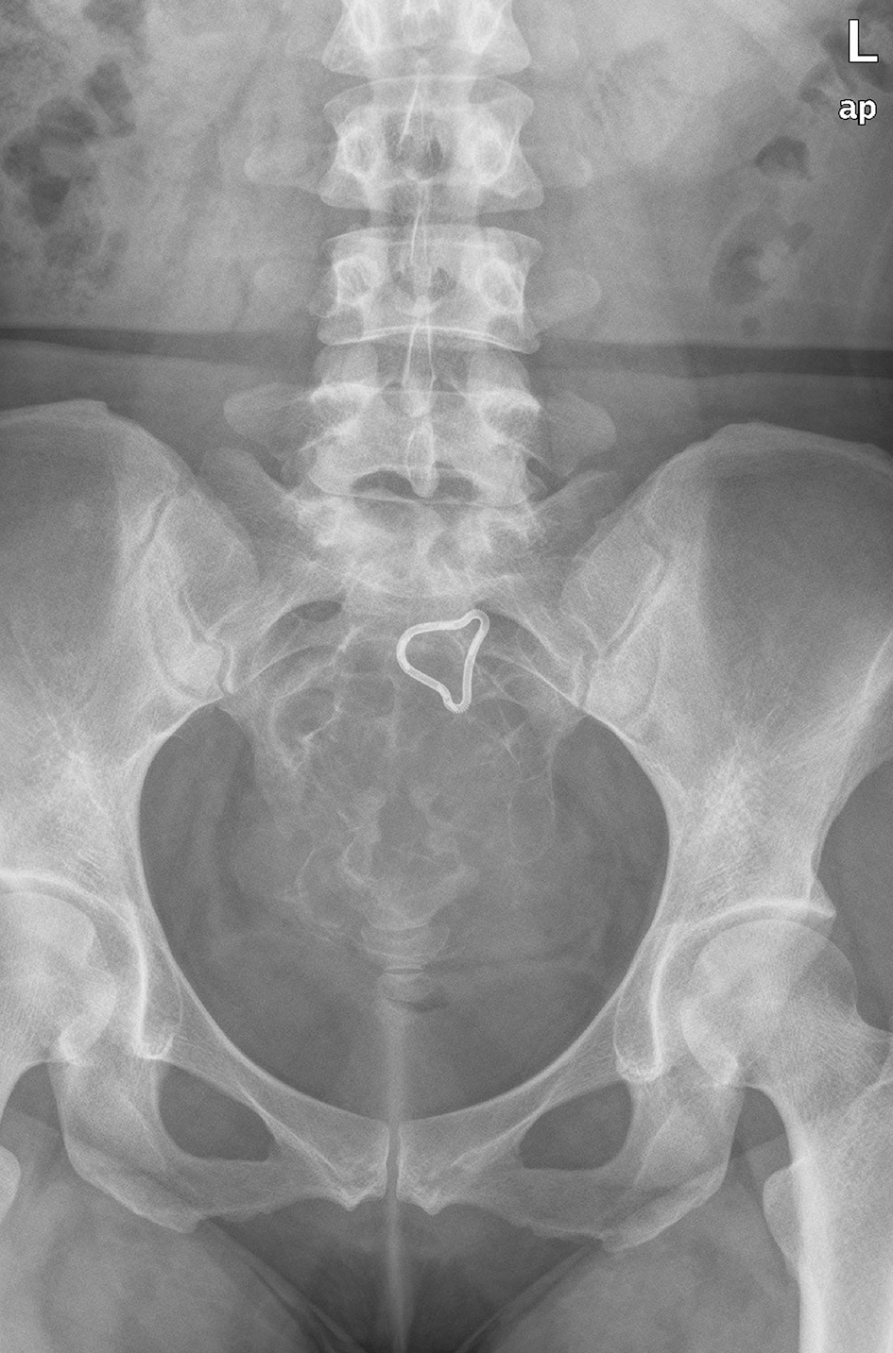
Radiograph shows markedly expansile, well-marginated, mixed lytic and sclerotic lesion of the sacrum.

**Figure 2.**
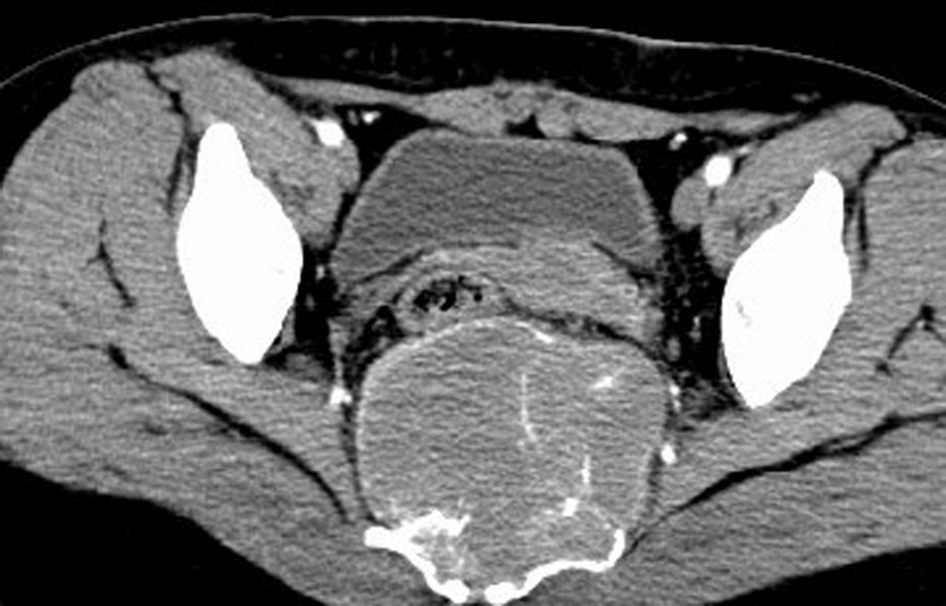
Contrast-enhanced axial CT shows a blood-riched, well defined, expansile, intraosseous tumor without cortical interruption in the sacrum. Some dense, coarse, trabeculated-like bones and residual bone crests within the tumor are observed. Extraosseous involvement of the soft tissue is absent.

**Figure 3.**
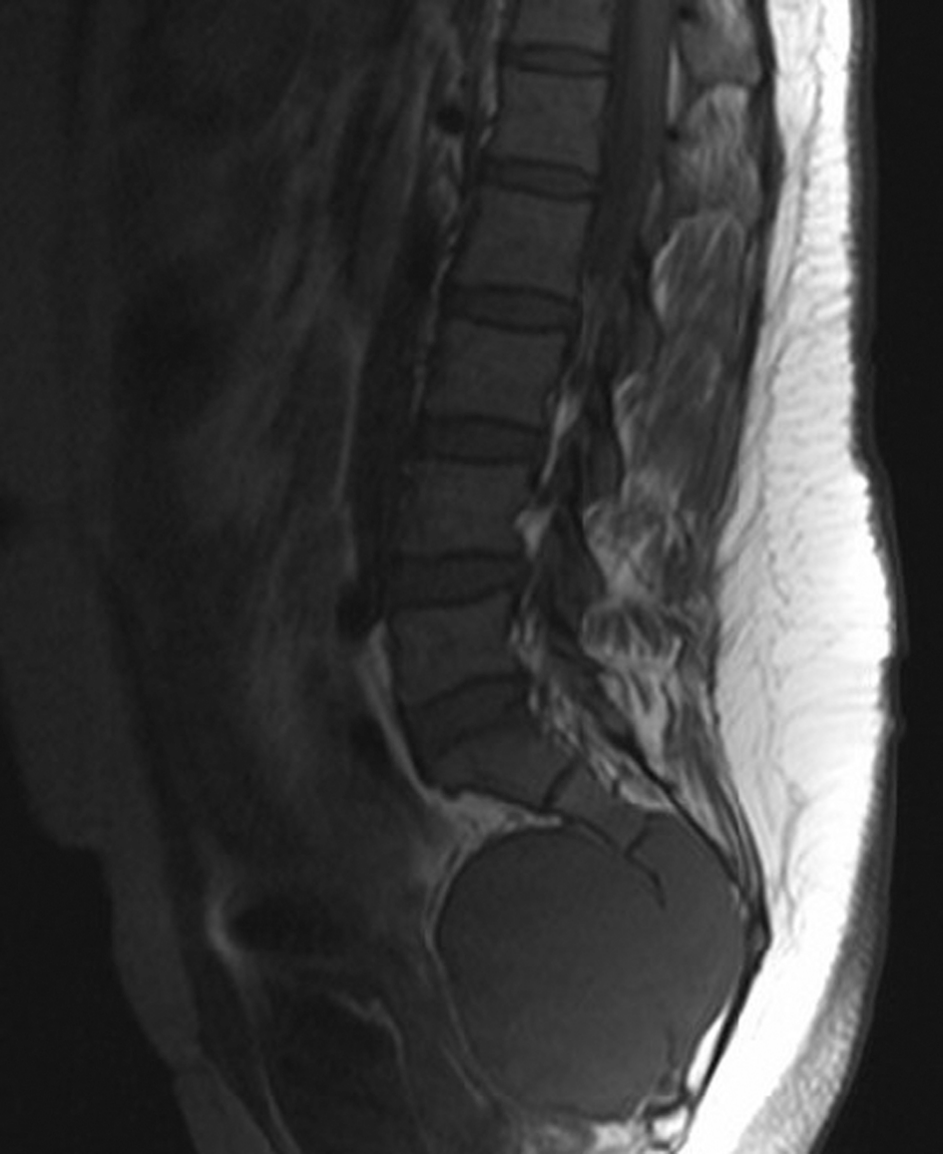
Sagittal MR T1-weighted imaging shows an expansile, well defined, homogeneously low signal mass with thinning cortex.

**Figure 4.**
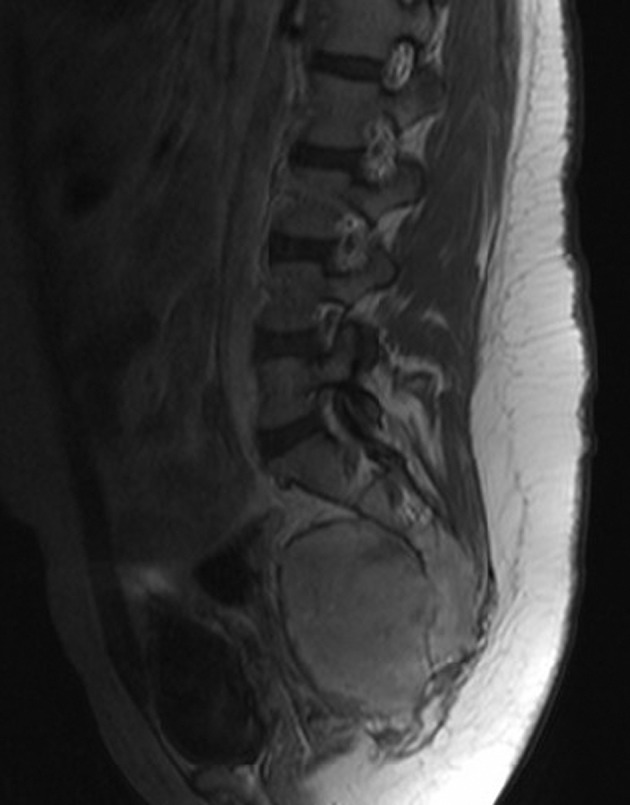
Contrast-enhanced sagittal MR T1-weighted imaging shows marked enhancement of the mass.

A 32-year-old woman was admitted to our hospital for a low echo-level mass searched by ultrasound in the routine physical examination 4 days before. The patient was asymptomatic. A full blood examination gave values within the normal limit. Plain film showed cortical thinning and discontinuity at the sacrum without reactive periosteal bone formation (Figure 1). Contrast-enhanced CT and MR were subsequently performed. CT revealed a round-like, well-marginated, soft-tissue mass in the sacrum bone (Figure 2). The tumor was expansible and no cortex was interrupted. Dense, coarse, trabeculated-like calcification or ossification within the tumor was observed. Extraosseous involvement of the soft tissue was absent. After injection of contrast medium, the mass was notably enhanced. Radial bone spicules and Codman triangle, which were viewed as characteristic features of malignancy, were also not found. MRI of the sacrum (Figure 3) showed a well-defined soft tissue mass with homogeneous low signal feature in T1-weighted imaging and high signal feature in T2-weighted imaging. Contrast-enhanced MR in T1-weighted imaging (Figure 4) displayed a markedly high signal feature suggesting rich blood supply of the tumor. Extraosseous involvement of the soft tissue was also not observed. 

**Figure 5.**
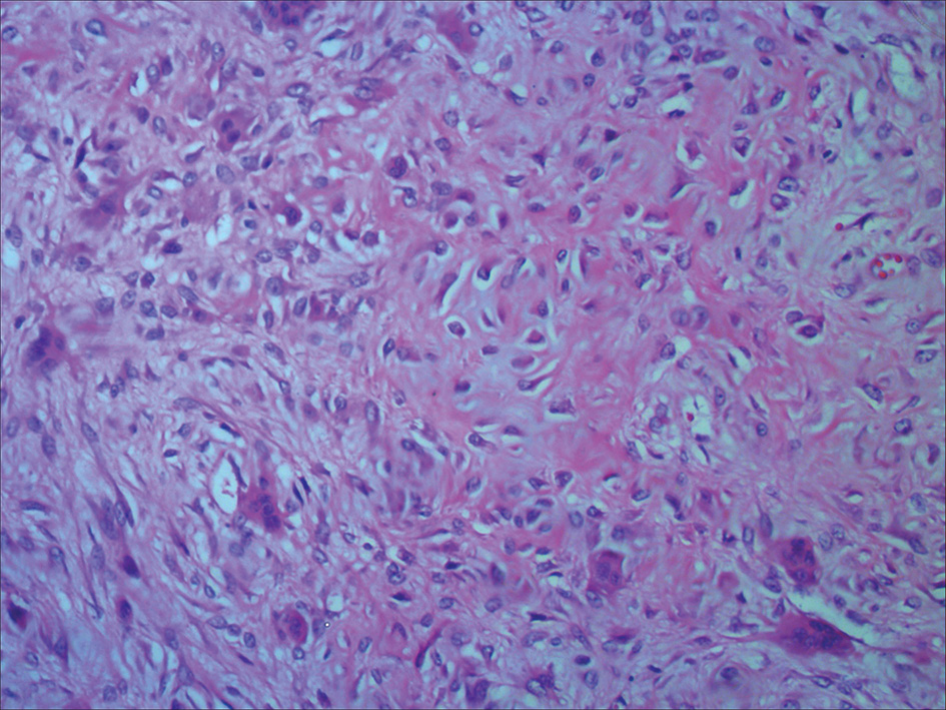
The spindle cells between the bony trabeculae show minimal cytologic atypia (HE, ×200).

The tumor was resected completely and successfully. Pathological diagnosis was well-differentiated intraosseous osteosarcoma. Histologically, the tumor was composed of newly formed irregular bony trabeculae and hypocellular spindle cells with only minimal atypia in the fibrous stroma (Figure 5). Twelve months after operation, the patient is doing well and there is no evidence of recurrence and distal metastasis. 

## 3. Discussion

Most osteosarcomas are highly malignant and hence, cytologically obvious. However, well-differentiated osteosarcoma is rare (accounting for approximately 1% of all osteosarcoma) and has a more benign, indolent course with less potential for metastasis and a higher survival rate compared with classic osteosarcoma (2, 4). This tumor, although malignant, may be mistaken for a benign condition, such as fibrous dysplasia. In a study of 80 well-differentiated osteosarcomas from the Mayo Clinic (5), local excision was almost always associated with recurrence. Wide excision was almost never followed by recurrence. The recurrent tumor was a high-grade, conventional osteosarcoma in 15% of the patients. Therefore, it is very important to distinguish this tumor from benign lesions and high-grade osteosarcomas.

Patients with well-differentiated osteosarcoma tend to be older than those with conventional osteosarcoma. The mean age for well-differentiated osteosarcoma is in the third decade(5). In this case, the patient was 32years old. There is no sex predilection. The distal femur and upper tibia are the most common sites of involvement (3). In our case, the tumor was located at an unusual site of the sacrum, which has not previously been reported in English literatures.

In our case, the tumor margin was well defined and no cortical disruption was observed. In 18 patients with well-differentiated osteosarcoma, Unni et al. (1) noted that the tumor margins were well-defined in two and poorly defined in 16, and showed cortical destruction in 14. Ellis et al. (3) reported eight cases of which five tumors were poorly-marginated and three tumors were well-marginated. Cortical interruption was identified in five of six other reported cases (6-9). Cortical disruption is unusual feature of fibrous dysplasia, unless a fracture has occurred. The tumor in our case was expansile and had cortical thinning that was a common finding in other series as well (1-3). Calcification or ossification within the tumor was observed in our case. We assume that lower grade lesions tend to grow more slowly and are differentiated enough to produce a dense, coarse, trabeculated-like bone. However, it is possibly more likely that part of this calcification and ossification is due to the incomplete destruction of residual normal bone or bone crest by tumor. The higher-grade lesions progress more rapidly and produce a tumor bone that is homogeneous and cloudlike or solid. Active periosteal response and extraosseous involvement of the soft tissue were absent in our case. Although this is a nonspecific finding by itself, when seen with increased bone density, radial bone spicules or Codman triangle, cortical destruction, and soft-tissue invasion, it is highly suggestive of osteosarcoma.

In summary, we reported a case of well-differentiated osteosarcoma of an unusual location of the sacrum. Correct diagnosis of well-differentiated osteosarcoma probably affects the decision of local excision or wide excision and the prediction of the recurrence and the survival rate. We consider that dense trabeculated-like bone within an intraosseous solid mass might be suggestive of well-differentiated osteosarcoma that was valuable in guiding the treatment and prediction of prognosis.

## References

[1] Unni KK (1996). Dahlin's Bone Tumors: General Aspects and Data on 11,087 Cases..

[2] Unni KK, Dahlin DC, McLeod RA, Pritchard DJ (1977). Intraosseous well-differentiated osteosarcoma.. Cancer..

[3] Ellis JH, Siegel CL, Martel W, Weatherbee L, Dorfman H (1988). Radiologic features of well-differentiated osteosarcoma.. AJR Am J Roentgenol..

[4] Dahlin DC, Unni KK (1977). Osteosarcoma of bone and its important recognizable varieties.. Am J Surg Pathol..

[5] Kurt AM, Unni KK, McLeod RA, Pritchard DJ (1990). Low-grade intraosseous osteosarcoma.. Cancer..

[6] Campanacci M, Bertoni F, Capanna R, Cervellati C (1981). Central osteosarcoma of low grade malignancy.. Ital J Orthop Traumatol..

[7] Sundaram M, Herbold DR, McGuire MH (1986). Case report 370.. Skeletal Radiol..

[8] Lodwick GS (1981). Case report 169: low-grade osteosarcoma of the tibia.. Skeletal Radiol..

[9] Unni KK (1981). Case report 136. Central low-grade osteosarcoma of tibia.. Skeletal Radiol..

